# Object localization with electrosensory mechanism in weakly electric fish

**DOI:** 10.1186/1471-2202-12-S1-P302

**Published:** 2011-07-18

**Authors:** Miyoung Sim, DaeEun Kim

**Affiliations:** 1 Biological Cybernetics, School of Electrical and Electronic Engineering, Yonsei University, Seoul, South Korea

## 

Weakly electric fish generate an electric field with their electric organ and detect the potential perturbation caused by a target object. There have been many studies of electrolocation in weakly electric fish, but the electrolocation rules have been suggested to reflect the biological experiments [1,2]. Modeling the electrosensory mechanism of weakly electric fish, we suggest an electrolocation method using the electric field potential. The discharge organ of the fish is typically located in the tail, and a large number of electroreceptors are distributed on the skin surface. A collection of electroreceptors along the rostrocaudal direction can measure the electric potential perturbed by a target object. Along the rostrocaudal line, we measure the normal and tangential components of the perturbation potential. If a few electric poles are sparsely distributed, the tangential component needs to be monitored. If one positive pole is available at the head and the other negative pole at the tail, the electric field direction is almost parallel to the rostrocaudal line. In fact, weakly electric fish have many positive poles distributed along the midline [2-4], and the normal component is more dominant in the middle of the body surface. With the normal components of the electric field potentials, object localization can be obtained.

We can theoretically derive an electrolocation rule that the sensor locations with maximum and minimum peak amplitude in the bimodal electric image, or the distance between the point with minimum peak amplitude and zero crossing point can determine the lateral distance of a target object. The maximum peak position approximately matches the rostrocaudal position of the object. This result matches with the result of other electrolocation rules [2,3]. Also we found the rule is qualitatively similar to the distance estimation with the ratio of maximum slope to maximum amplitude suggested by von der Emde [1]. The electrolocation rule can be modeled with a possible neural system.
